# Hydration-Controlled Proton Transport in Respiratory Complex I

**DOI:** 10.1021/jacs.5c22499

**Published:** 2026-03-27

**Authors:** Jong Ho Choi, Gregory A. Voth

**Affiliations:** Department of Chemistry, Chicago Center for Theoretical Chemistry, James Franck Institute, and Institute for Biophysical Dynamics, The University of Chicago, Chicago, Illinois 60637, United States

## Abstract

Proton pumping by respiratory complex I is one essential element for generating the proton motive force that drives ATP synthesis in mitochondria. Although it is understood that electrons from NADH reduce ubiquinone at the peripheral arm and that four protons are transferred in the membrane domain, the mechanism by which this redox reaction initiates proton translocation remains unclear. A lateral pathway linking the quinone binding site to the membrane domain via ND1, ND3, and ND4L subunits has been proposed as a possible initial path of an excess proton. However, experimental structures indicate that the hydration connectivity between D66_ND3_ and E34_ND4L_ is comparatively weaker than in neighboring segments, suggesting a potential regulatory point for proton transfer. Using multiscale reactive molecular dynamics (MS-RMD) and a water wire connectivity metric, we directly simulate proton transport through this region as coupled to the hydration by water molecules. Our results reveal that proton transfer is thermodynamically feasible when transient hydration aligns with the presence of an excess proton, revealing the strong coupling between hydration and proton transfer (PT) in this region of Complex I. These findings support a model where proton injection enhances local hydration, dynamically opening the pathway for proton transfer and regulating the onset of proton pumping in Complex I.

## Graphical abstract

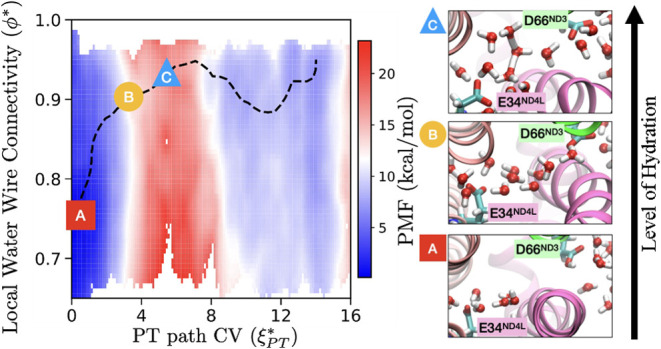

## Introduction

Respiratory complex I (NADH: ubiquinone oxidoreductase) is the largest enzyme in the electron transport chain (ETC).^1–5^ During its catalytic cycle, two electrons from NADH oxidation are transferred through a chain of Fe–S clusters and reduce ubiquinone, and the free energy released in this redox reaction is harnessed to translocate four protons across from N-side to P-side, contributing to the proton motive force that ultimately drives ATP synthesis.^5–11^ Complex I is a massive protein cluster, approximately 200 Å in length, composed of a hydrophilic peripheral arm and a membrane domain (Figure 1). A central question regarding this enzyme is how electron transfer and redox reaction that occurs in the peripheral arm is coupled to proton pumping in the membrane domain, a process thought to involve both large-scale conformational changes and local proton-transfer pathways.

**Figure 1:**
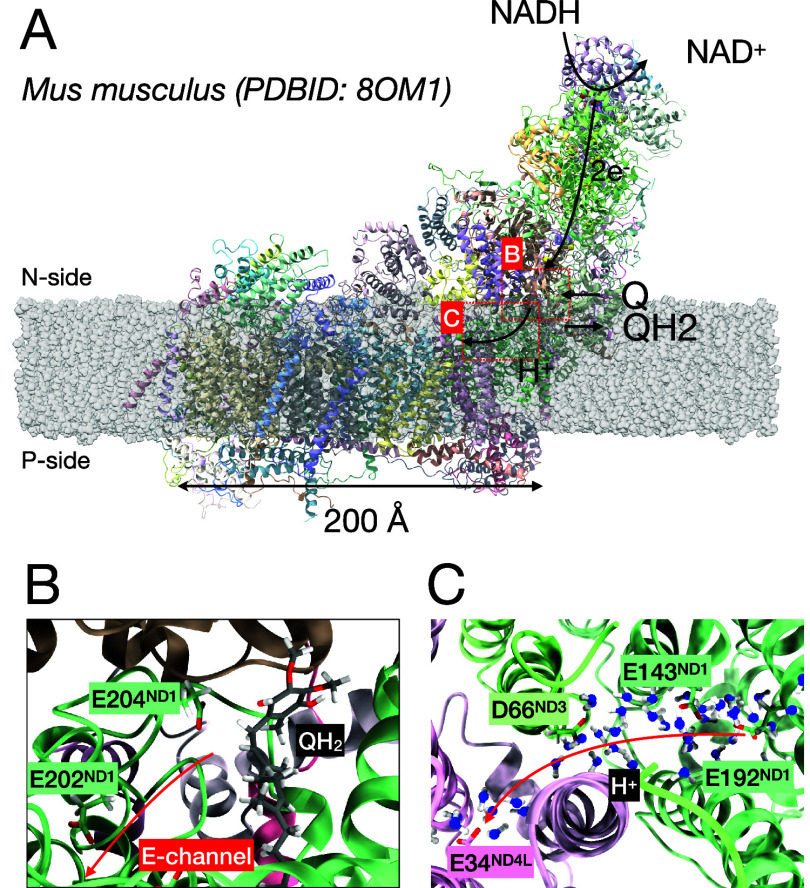
(A) The structure and working mechanism of active *Mus musculus* Complex I. (PDBID: 8OM1) (B) The configuration of ubiquinol (QH_2_) and the entrance of E-channel. The arrow indicates a suggested pathway for proton injection into the E-channel. (C) The structure of water wire inside the PT channel through E192_ND1_ to E34_ND4L_. The oxygens of water molecules are represented as blue for easier comparison to the oxygens of GLU and ASP. The snapshot is rotated to provide a view from the N-side toward the P-side and to more clearly visualize the arrangement of water molecules.

Recent structural studies have provided critical insights into these processes by capturing the enzyme in distinct conformational states, broadly categorized as “closed” (or active) and “open” (or deactive) forms.^12–18^ These transitions are defined by the ordering or disordering of conserved loops surrounding the ubiquinone-binding cavity and, crucially, by structural rearrangements within the membrane domain, such as the formation of a π-bulge in transmembrane helix 3 of subunit ND6.^18–20^ This specific structural element has been proposed to act as a gate that modulates the water connectivity of the “E-channel,” a hydrated pathway linking the redox-active site to the membrane interior.^20–22^ Mechanistically, this gating is intimately linked to the dynamics of ubiquinone, which is thought to shuttle between a reduction site near iron–sulfur cluster N2 and a second site proximal to the E-channel.^9,14,23^ Current models diverge on how this drives proton pumping: some propose that redox-induced formation of anionic quinol species triggers what has been described as an “electrostatic wave,” promoting proton injection into the membrane domain,^4,21,24^ while others describe a “domino-like” mechanism in which protons are taken from the E-channel to protonate the quinone, thereby initiating proton pumping.^16,17^ Regardless of the specific coupling model or the physiological role of the open conformation, there is growing consensus that dynamic hydration within these conserved channels is fundamental to the long-range coupling mechanism.^20,21,25^

Building on the view that multiple mechanistic models may operate within the structural framework, most proposals agree that proton transfer must be initiated within the “active” (closed) state of complex I, where hydrogen-bonded water networks are required to support proton pumping.^16,18^ Consistent with this picture, recent high-resolution cryo-EM studies of the active enzyme have resolved ordered water molecules lining putative proton-transfer pathways.^19,20^ However, determining whether these observed water arrays are quantitatively sufficient to support proton transfer remains challenging, because static snapshots captured at cryogenic temperatures cannot fully account for hydration fluctuations or the dynamic connectivity at physiological temperatures.^25,26^ This limitation is particularly acute at the interface between ND1 and ND3/ND4L subunits within the E-channel, a critical junction for proton delivery. In the high-resolution structure of the active mammalian Complex I (PDB 8OM1), resolved water molecules between the conserved residues D66_ND3_ and E34_ND4L_ exhibit a separation of ∼4.5 Å along the putative proton-transfer pathway.^27^ This region therefore emerges as a potential kinetic bottleneck, where the fluctuation of the water network may regulate the rate of proton transfer, yet the energetics and dynamics of hydration across this gap remain poorly understood.^5,10^

Several previous simulation studies have investigated hydration patterns and possible proton transfer pathways in the ND1–ND4L region using classical molecular dynamics and Quantum Mechanics/Molecular Mechanics (QM/MM) methods.^25,28–32^ These efforts typically assessed hydration by counting the number of water molecules within the pathway. While such metrics offer useful structural insight, they do not quantify the continuity of the water network or capture the dynamic role of hydration in proton translocation.^33^ Moreover, because proton transport (PT) involves bond breaking and formation as an excess proton shuttles between water molecules and (at times) protonatable amino acids, it cannot be modeled with traditional nonreactive or “classical” MD simulations. Although the QM/MM method can describe bonds breaking and forming, because of its demanding computational cost, QM/MM simulations are limited to several picosecond time scales, making them inadequate for capturing much slower hydration dynamics and its coupling to proton transfer. A common practice in QM/MM simulations of PT processes in proteins such as Complex I, therefore, is to first use standard nonreactive MD runs to capture what appear to be favorable hydrating water structures and then to utilize QM/MM with umbrella sampling over a number of umbrella windows to sample the PT. This approach is to estimate the so-called potential of mean force, or PMF, which is the free energy profile of the excess proton migration along some defined reaction coordinate or “collective variable (CV)”. However, there are two limitations with this approach. The first is that the water hydration is generally explicitly coupled to the presence of a excess proton in a cooperative fashion and this is largely neglected.^33^ (As will be shown later, our simulations indicate that the rearrangement of water molecules in response to an excess proton can require several hundred picoseconds.) Second, the time scale of the QM/MM sampling in the umbrella windows is generally just a few picoseconds per window given the large computational cost, but the proton hopping between just two waters occurs on that same time scale (∼2 ps in liquid water) and so the QM/MM statistical sampling with such short time scales would be far from complete. (It is not uncommon, e.g., with more efficient reactive MD simulations, to sample in the multiple nanosecond range per umbrella window.^33–39^ )

To overcome the limitations of using traditional MD combined with QM/MM as described in the previous paragraph, we have employed multiscale reactive molecular dynamics (MS-RMD)^34^ with explicitly protonatable amino acids. This simulation framework enables explicit modeling of PT via the Grotthuss shuttling mechanism with dynamic protonation of amino acids and has been used extensively to study proton transfer in numerous biomolecular systems.^33,35–45^ In MS-RMD, proton transport is described by constructing a multistate Hamiltonian spanning different protonation configurations and diagonalizing it on-the-fly, allowing continuous proton delocalization and transfer over extended time scales. This approach extends the accessible time and length scales of reactive simulations over QM/MM by 2–3 orders of magnitude, enabling us to capture how complex hydration dynamics are coupled to and influence the PT. To quantitatively characterize the hydration, we further applied water wire connectivity order parameter or collective variable (CV), which quantifies the connectivity of water molecules and amino acids along a PT pathway.^33^ Because the water wire connectivity is formulated as a smooth and continuously differentiable function of atomic coordinates, it can be efficiently biased in enhanced sampling frameworks, allowing systematic exploration of hydration-coupled proton transfer. Together, these methods provide a powerful platform for probing the interplay between hydration dynamics and proton translocation in Complex I.

In this study, we performed MS-RMD simulations for the active *Mus musculus* Complex I system to investigate proton transfer from the E-channel toward the membrane domain. We focused on the PT pathway spanning E192_ND1_ through D66_ND3_ to E34_ND4L_. To track the location of the excess proton along this path, we applied a curvilinear PT path collective variable (ξ*_PT_), and to quantify the hydration environment surrounding the proton, we used a water wire connectivity (ϕ).^33^ Here, ξ*_PT_ is a unitless variable that reports the location of the excess proton along a predefined curvilinear pathway from E192_ND1_ to E34_ND4L_, while ϕ is a unitless variable ranging from 0 to 1 that quantifies the degree of connectivity of water molecules between predefined nodes along the channel. The exact definition of both collective variables (CVs) can be found in the Method Section and the Supporting Information. Consistent with previous studies, we found that the segment between D66_ND3_ and E34_ND4L_ exhibits intermittent and fluctuating hydration in the absence of an excess proton. However, our free energy analysis based on the two CVs reveals that PT across this region is not only thermodynamically accessible, but also coupled to the local hydration, highlighting the role of dynamic water networks in regulating proton translocation.

## Results and Discussion

### Classical MD Simulation and Water Wire Connectivity Analysis

We obtained a stable *Mus musculus* Complex I structure after 1 μs of classical MD simulation. Figure 1A illustrates the equilibrated structure of Complex I embedded in a lipid membrane (the composition and properties of which are given in the Methods section), along with a schematic representation of its overall mechanism. Electron transfer through the chain of iron–sulfur clusters leading to the reduction of ubiquinone is a well-established process.^1,2^ In contrast, how this redox event is coupled to proton transfer into the E-channel remains under active investigation, with multiple mechanistic models proposed.^4,16,24^ Across these models, there is general agreement that the reduced quinone does not remain static at the primary reduction site near cluster N2 but relocates within the quinone-binding cavity.^9,17,20,21,23,25^ Specifically, some mechanistic studies propose that migration of reduced quinone toward a distal position within the quinone-binding cavity provides a thermodynamic contribution to proton pumping,^10,23^ whereas other studies propose that proton pumping is initiated during the quinone reduction process itself, and that the subsequent relocation of quinone occurs after full reduction, as part of the conformational resetting of the enzyme.^16,17^ Motivated by proposals from multiple mechanistic models that, following quinone reduction, QH_2_ can occupy positions closer to the entrance of the E-channel, we modeled QH_2_ near the E-channel entrance as a representative postreduction configuration, rather than to reproduce a specific experimentally resolved binding site. We confirmed that its position is stable during the simulation without explicit restraints. Figure 1B presents the configuration of the QH_2_ and the entrance region of the E-channel from our simulation.

In our simulations, a water network was observed along the region from QH_2_, through the E-channel, to the ND4L subunit. Figure 1C shows a representative snapshot of the water network, spanning from E192_ND1_ through D66_ND3_ to E34_ND4L_. This observation is broadly consistent with previous experimental^20,26,28,46,47^ and computational studies;^25,29^ however, a key advance of this work is the ability to quantitatively assess the hydration of the D66_ND3_–E34_ND4L_ region–previously identified as relatively weakly connected–through thermodynamic analysis of water wire connectivity (ϕ). Note that we use the water-wire connectivity (ϕ) that is averaged along the entire path, as these simulations are nonreactive and contain no excess proton. The path for calculating ϕ was constructed from the simulation trajectory by applying a principal curve algorithm^48^ to the positions of water oxygen atoms. The path is represented as 25 equidistant nodes which are shown in Figure 2A. To capture the thermodynamics of the hydration, we ran a set of multiple walkers well-tempered metadynamics simulations^49^ with biasing ϕ. As shown in Figure 2B, the potential of mean force (PMF, i.e., conditional free energy path) of ϕ has a broad well ranging from 0.5 to 0.8. Its minimum is at ϕ = 0.67 (which signifies an incomplete hydration), and there are metastable states ϕ = 0.85 and ϕ = 0.45. Figure 2C shows representative snapshots at two distinct levels of ϕ. Although the ϕ values differ by more than 0.4 between the two states, the overall arrangement of water molecules remains similar, with a few specific water molecules between D66_ND3_ and E34_ND4L_ exhibiting differences. This indicates that the ϕ is highly sensitive to the arrangement of a few key water molecules in a small region.

**Figure 2:**
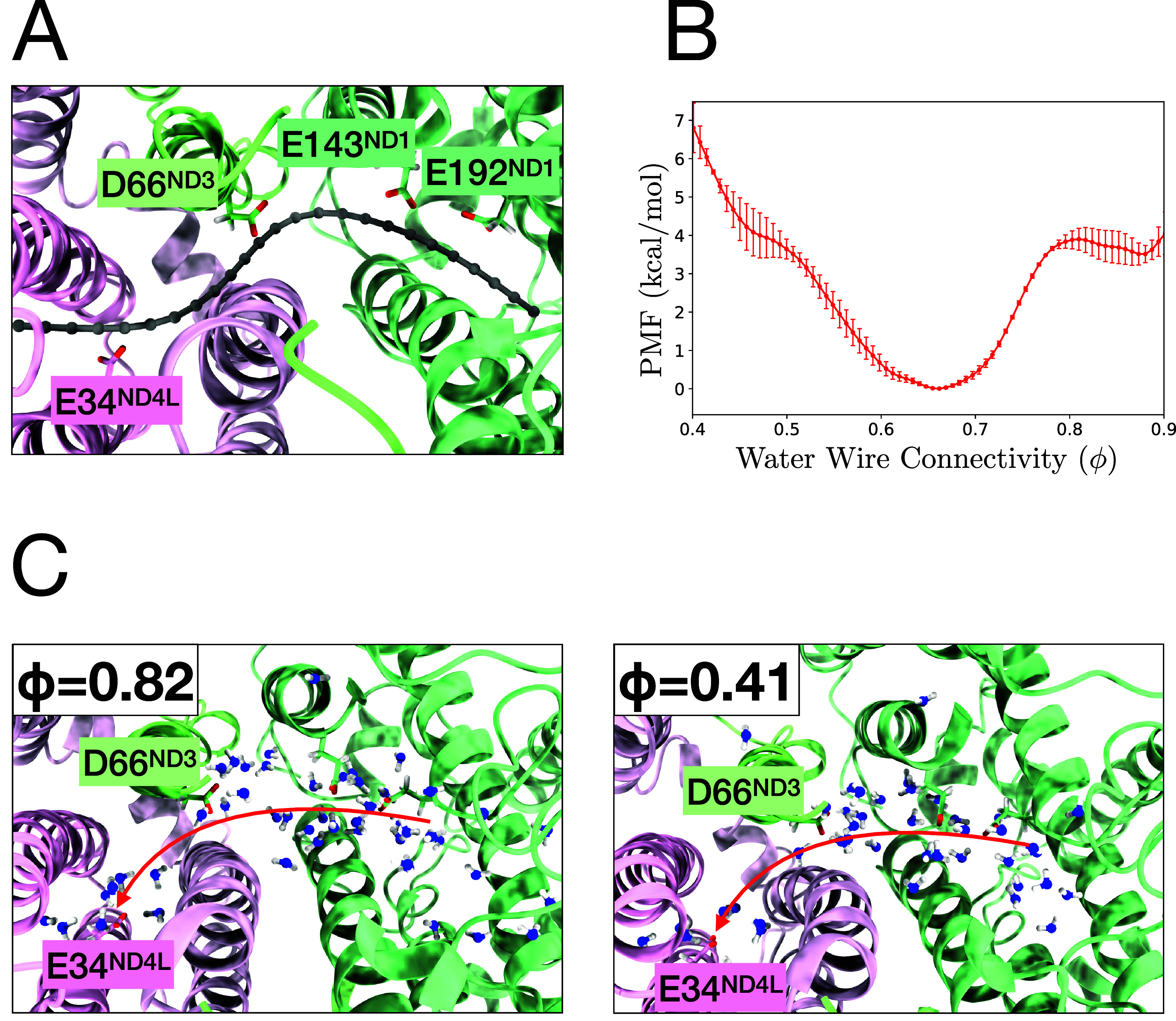
(A) A representative snapshot showing the positions of nodes used to compute water wire connectivity. (B) PMF for water wire connectivity (ϕ) obtained from multiple walkers well-tempered metadynamics simulations. (C) Representative snapshots illustrating the difference in water molecule arrangements at ϕ of 0.82 and 0.41. Arrows indicate a suggested pathway for proton transport.

Based on these observations, we divided the sampling of the actual PT pathway into two segments and applied umbrella sampling (US) separately for each. For the D66_ND3_–E34_ND4L_ region, which exhibits intermittent and fluctuating hydration, we performed two-dimensional US using two CVs: the position of the excess proton along the curvilinear PT path (ξ*_PT_) and the local water wire connectivity (ϕ*), allowing us to map the free energy surface of the PT reaction. Note that ϕ* is a localized version of water wire connectivity ϕ that quantifies the organization of water molecules near the excess proton by selectively weighting the contribution of nodes near the excess positive charge. In contrast, for the stably hydrated E192_ND1_–D66_ND3_ region, we employed one-dimensional US biasing ξ*_PT_ to compute the PMF as the water connectivity is not an issue in that region. To distinguish these two segments easier, we define ξ*_PT_ by setting the node closest to D66_ND3_ as the origin (ξ*_PT_ = 0), with positive values pointing toward E34_ND4L_ and negative values toward E192_ND1_. Although ξ*_PT_ is unitless, it is rescaled to yield values that are numerically comparable to distances in Å.

### 2D-Umbrella Sampling MS-RMD Simulations for D66-E34

The 2D-PMF of PT in D66_ND3_–E34_ND4L_ region is presented in Figure 3A. The dashed line shows the minimum free energy path (MFEP) of the PT reaction. Note that each umbrella sampling window was sampled for 1–2 ns, and the resulting PMF was averaged over the last four out of eight equally divided blocks. Notably, the PMFs from the first 4 blocks showed significant deviation from the final PMFs, indicating that at least 0.5–1 ns of simulation per US window was required for the convergence of the PMF (Figure S1), which is well beyond the statistical sampling presently accessible by any sort of QM simulation. This result indicates that several hundred picoseconds are needed for water molecules and the excess proton to reorganize within each window and highlights a limitation of QM/MM methods that rely on only a few picoseconds of sampling per umbrella window: This is not enough sampling for the necessary equilibration of water and proton configurations. Even if long classical MD simulations were used to first equilibrate certain water configurations, the presence of an excess proton alters the local hydration thermodynamics.

**Figure 3:**
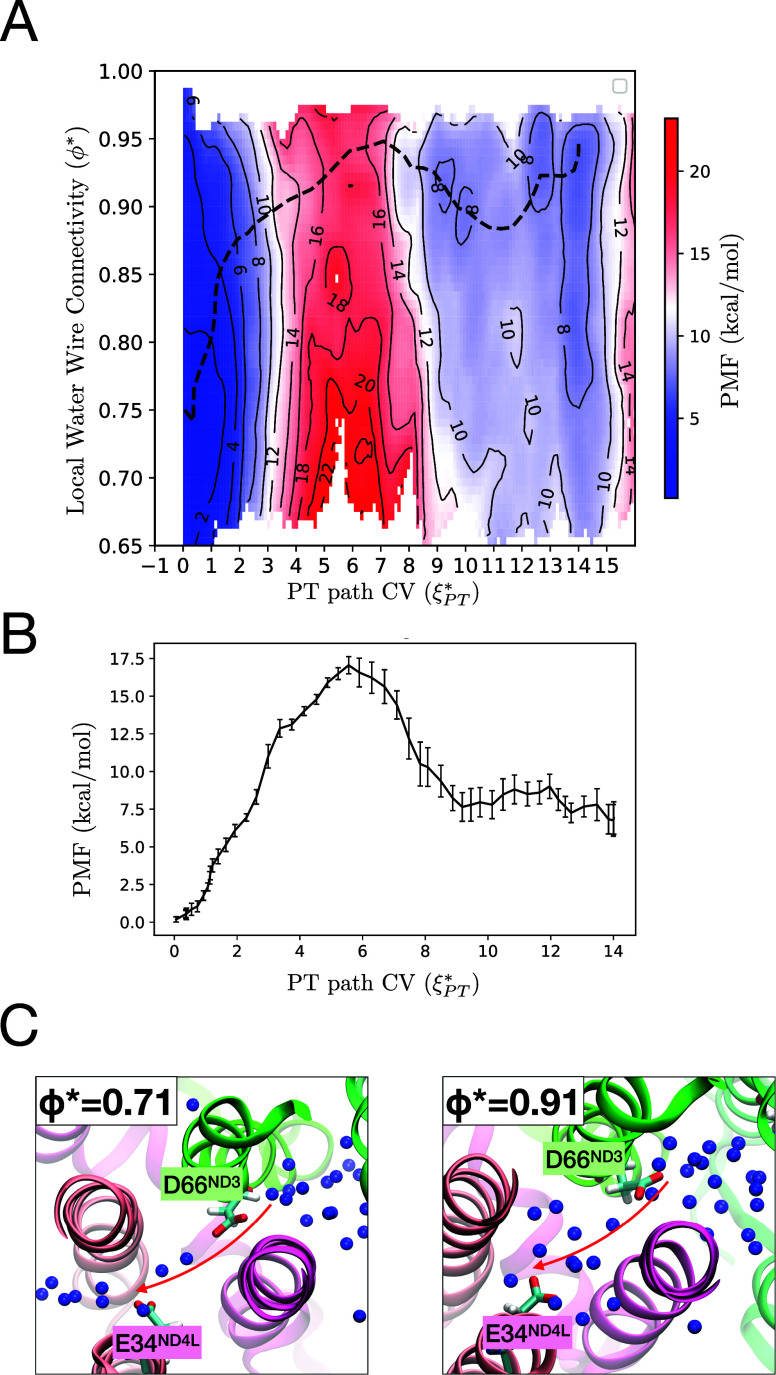
(A) Two-dimensional potential of mean force (2D-PMF) as a function of the local water wire connectivity (ϕ*) and PT path CV (ξ*_PT_), obtained from 2D umbrella sampling. The minimum free energy path (MFEP) is shown as a dashed line. (B) The PMF of the MFEP projected along the ξ*_PT_. (C) Representative snapshots of water molecule arrangements from two windows at ξ*_PT_ = 5.5, each exhibiting distinct ϕ*, 0.71 and 0.91. Arrows indicate a suggested pathway for proton transport.

The 2D PMF reveals a coupling between the two CVs in the region near D66_ND3_, where ξ*_PT_ ranges from 0 to 5. When ξ*_PT_ = 0, corresponding to a protonated D66_ND3_, the free energy minimum is at ϕ*≈ 0.75. In contrast, at the saddle point around ξ*_PT_ ≈ 6, ϕ* exceeds 0.9, indicating a highly connected water chain. The MFEP (dashed line) shows that as ξ*_PT_ increases from 0 to 5, ϕ* also increases, indicating explicit coupling between the proton position and hydration^33^–i.e., as the excess proton moves forward, the less hydrated regions become more easily hydrated. Such proton-induced hydration has been commonly observed in previous studies of other proton channels.^33,35,39,41,50,51^ Although multiple large-scale regulatory mechanisms, including quinone reduction, quinone migration, and open/closed conformational transitions, have been proposed to regulate proton pumping in Complex I, our results indicate that, at the level of the D66_ND3_–E34_ND4L_ segment, coupling between proton position and local hydration constitutes an important factor governing proton transfer across this critical region.

Figure 3B shows the PMF of the MFEP projected on ξ*_PT_, indicating an endergonic proton transfer reaction with an energy barrier (Δ*F* ^‡^) of 17.1 ± 0.6 kcal/mol and a reaction free energy (Δ*G*) of 7 ± 1.0 kcal/mol. Using the classical transition state theory expression, $k=\frac{k_{B}T}{h}\exp\left(-\frac{\Delta F^{‡}}{\mathrm{RT}}\right)$ , a rough estimate of the proton transfer rate yields 6.1 s^–1^. Although this barrier is moderately high, it is comparable to the energy released upon ubiquinone reduction (∼800 meV = 18.5 kcal/mol). Moreover, as shown in Figure 4, the E192_ND1_–D66_ND3_ proton transfer is exergonic, further supporting the idea that the overall PT is feasible. In contrast, the 2D PMF allows us to estimate the barrier under low hydrated conditions. When ϕ* = 0.7, the barrier height increases to 22 kcal/mol and the estimated reaction rate is 0.002 s^–1^. This estimated high barrier indicates that proton transfer from D66_ND3_ to E34_ND4L_ is strongly suppressed in less hydrated environments.

**Figure 4:**
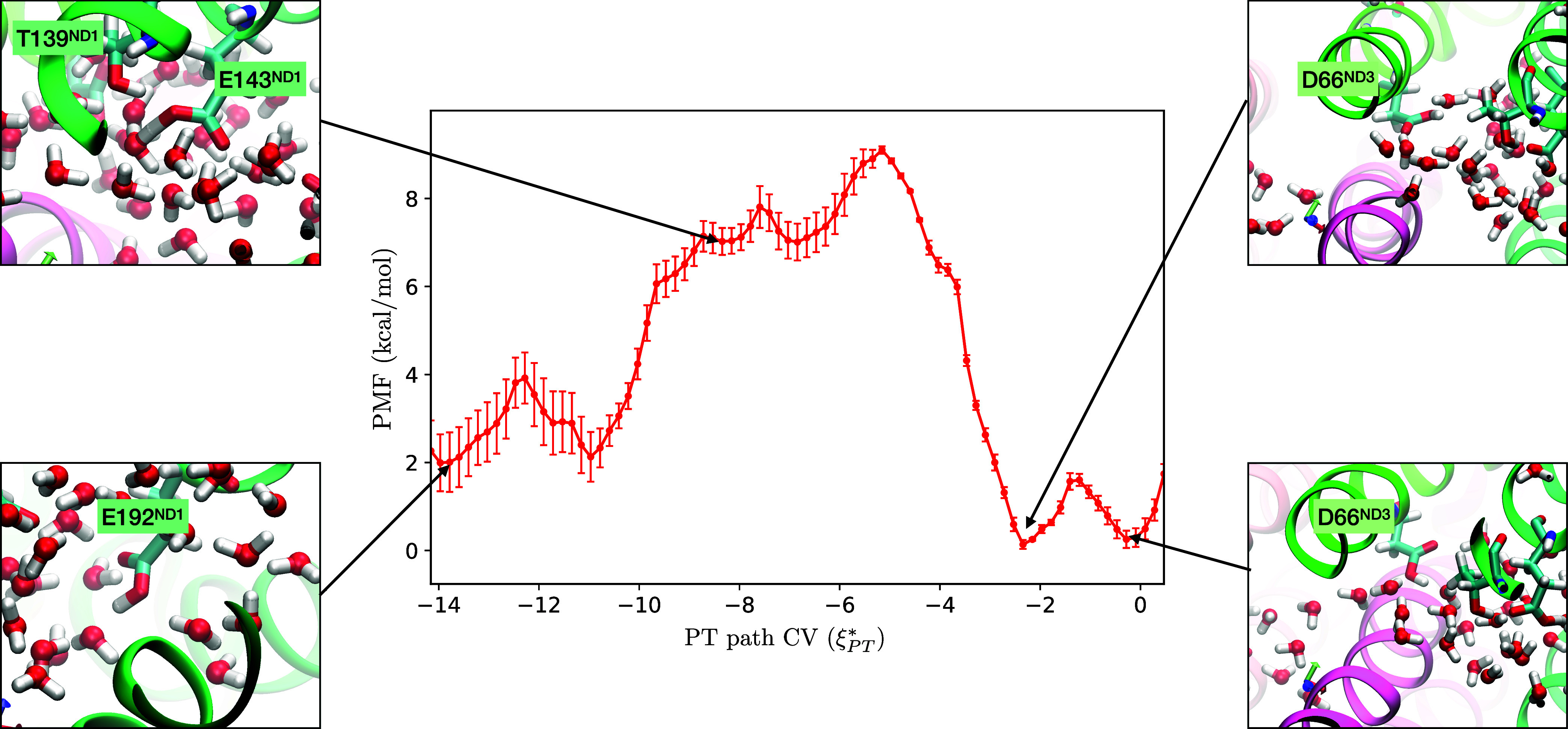
One-dimensional potential of mean force (1D-PMF) for proton transfer along the E192_ND1_–E143_ND1_–D66_ND3_ pathway. Representative snapshots are shown for selected positions along the reaction coordinate, highlighting distinct proton positions and water molecule arrangements.

In a recent paper, Sharma et al. reported a QM/MM free-energy barrier of approximately 11 kcal/mol in the same region.^32^ While this similarly identifies a thermodynamic bottleneck, their calculation differs from ours in several important respects. Because of the limited time scale accessible to their QM/MM simulations, the surrounding water molecules remained effectively static, so the energetic cost associated with organizing the water network was not sampled. In addition, their D66_ND3_ side chain was oriented toward E34 at the start of the calculation, whereas our simulations show that D66_ND3_ initially points in the opposite direction. Thus, the rotational rearrangement of this residue—which contributes to the overall barrier in our system—was not captured in their free-energy profile. These methodological differences likely explain the quantitative disparity in the barriers while remaining qualitatively consistent.

Given the critical role of hydration in proton transfer, a quantitative descriptor such as water wire connectivity is essential. Figure 3C presents two representative snapshots at ξ*_PT_ ≈ 5.5 with differing ϕ* values. As shown in the left configuration, a few water molecules are aligning between D66_ND3_ and E34_ND4L_ even at less hydrated state (ϕ* = 0.71). In a typical structural assessment, which checks whether water molecules are present within a predefined distance, this configuration can be classified as hydrated state. However, our PMF reveals a significant free energy difference between the two states, and PT is unlikely to proceed in the lower- ϕ* case. By employing ϕ*, we were able to distinguish the thermodynamic disparity between these hydration states.

### 1D-Umbrella Sampling MS-RMD Simulation on E192-D66

Compared to the proton transfer from D66_ND3_ to E34_ND4L_, the transfer from E192_ND1_ to D66_ND3_ occurs more readily. As shown in Figure 4, the transfer from E192_ND1_ to D66_ND3_ is exergonic, with a free energy decrease of 2 kcal/mol. The activation barrier (7 kcal/mol) is much lower than 17.1 kcal/mol barrier of transfer from D66_ND3_ to E34_ND4L_, indicating rapid proton transfer to D66_ND3_. The PMF profile along ξ*_PT_ reveals distinct protonation states of residues across the pathway. In the range of ξ*_PT_ = −14 to −10, where E192_ND1_ is protonated, showing two clear local minima corresponding to the protonation of each of carboxylate oxygens. In the region between −2 and 0, D66_ND3_ is protonated as shown in the two right snapshots of Figure 4. In contrast, at ξ*_PT_ = −8 to −6, where the excess charge is near E143_ND1_, there are shallow and less distinct minima indicating that E143_ND1_ is not well protonated, unlike the other acidic residues. The transition state for proton transfer appears between E143_ND1_ and D66_ND3_ at ξ*_PT_ = −5.

A noteworthy finding is that E143_ND1_ is rarely protonated unlike the other Glu and Asp in this PT path. The left top snapshot of Figure 4 shows E143_ND1_ and surrounding water molecules at ξ*_PT_ ≈ −8. A hydronium ion is sufficiently close to protonate E143_ND1_, but its protonated state is not the lowest energy state in the MS-RMD method. Instead, T139_ND1_ donates its hydrogen in a hydrogen bond to E143_ND1_. Due to this hydrogen bond, the p*K*_a_ of E143_ND1_ decreases; consequently, making it less favorable to be protonated. Importantly, this protonation behavior and its underlying mechanism would not have been accessible through nonreactive MD simulations; it was only revealed by employing MS-RMD, which explicitly accounts for dynamic proton delocalization and transfer events. Interestingly, a recent study did not observe such an interaction between E143_ND1_ and T139_ND1,_ ^32^ most likely because E143_ND1_ was kept in its neutral form during classical MD simulations. However, E143_ND1_ is expected to be deprotonated during the PT process, and our results suggest that the effect of T139_ND1_ may further lower its p*K*_a_, making E143_ND1_ less likely to be in a neutral state than previously assumed.

Taken together, our MS-RMD simulations provide a quantitative free-energy and kinetic description of proton transfer within the E-channel toward the ND4L subunit in Complex I. For the first half of the pathway, from E192_ND1_ to D66_ND3_, the proton transfer is exergonic with Δ*G* = −2 kcal/mol and has barrier of 7 kcal/mol (Figure 4). For the other half from D66_ND3_ to E34_ND4L_, the proton transfer is endergonic with Δ*G* = 7 kcal/mol and has a significantly higher barrier (Figure 3B). The overall free energy increase for the PT (5 kcal/mol) is smaller compared to the energy release upon the reduction of ubiquinol (18.45 kcal/mol). However, the large free energy barrier between D66_ND3_ and E34_ND4L_ (17 kcal/mol) suggests that the D66_ND3_-E34_ND4L_ region can be a kinetic bottleneck of the PT for the first step of proton pumping in Complex I.

By using the water wire connectivity analysis, we showed that the proton transfer from D66_ND3_ to E34_ND4L_ is highly coupled with the local hydration in the channel, and that the presence of an excess proton can actively stabilize hydration and thereby facilitate transfer across this otherwise weakly connected region. Although the present simulations assume a postreduction state in which QH_2_ is already formed, alternative mechanistic models suggest that protons required for quinone reduction may originate from the E-channel or antiporter-like subunits such as ND2 or ND4L, implying proton motion in the opposite direction along the same pathway.^17^ Importantly, our umbrella sampling framework does not impose a preferred direction for proton transfer, allowing the same PMF results to be interpreted for reverse transfer along the E34_ND4L_–D66_ND3_ segment. In this context, proton transfer from E34_ND4L_ toward D66_ND3_ is associated with a substantially lower free-energy barrier (≈10 kcal/mol) than in the forward direction, and inspection of Figure 3A shows that when the excess proton is localized near E34, the surrounding hydration remains consistently high, indicating that hydration does not impose a significant additional barrier near the transition state. Together, these results indicate that hydration–proton coupling modulates proton transfer along the E-channel in a direction-dependent manner. While proton-induced hydration is critical for enabling transfer across the D66_ND3_–E34_ND4L_ region in one direction, sufficient local hydration near E34_ND4L_ renders the reverse transfer less hydration-limited.

In this context, our results indicate that hydration-coupled proton transfer within the ND1–ND3–ND4L segment represents a local regulatory element embedded within the broader coupling mechanism of Complex I. While global conformational transitions between open and closed states are known to modulate overall channel hydration, our simulations demonstrate that, even within the catalytically relevant closed state, proton translocation is governed by a finer-scale gating process in which the excess proton actively reshapes the local hydration environment. This microscopic hydration gating is compatible with multiple proposed coupling mechanisms: in electrostatic or proton-injection models, it provides a quantitative description of how protonic charge drives transient water-wire formation, whereas in conformational or domino-like models, it defines a specific kinetic bottleneck that must be overcome for energy to propagate into the membrane domain. Thus, rather than favoring a single mechanistic framework, our findings highlight dynamic wetting transitions as a common and necessary component of postreduction signal propagation through the E-channel.

In our MS-RMD simulations, the key acidic residues in the E-channel (E192_ND1_, D66_ND3_, and E34_ND4L_), which were modeled in their neutral state in the preceding classical MD simulations, were intentionally switched to their deprotonated (charged) states, allowing a single excess proton to move dynamically among them. This choice was made to enable explicit tracking of one transferable proton and thereby unambiguously resolve the correlation between proton position and local hydration, without complications arising from multiple interacting protons or fixed protonation states. Such a setup differs fundamentally from classical MD, where protonation states remain static, and is essential for capturing hydration–proton coupling along the transfer pathway. Although the predicted p*K*_a_ values of these residues (7.86 for E192_ND1_, 8.05 for D66_ND3_, and 7.62 for E34_ND4L_) suggest a tendency toward protonated states under standard conditions, partial deprotonation remains thermodynamically plausible given physiological pH variability and strong local electrostatic interactions. Importantly, a recent study by Uddin et al.,^52^ performed on a bacterial homologue of Complex I, demonstrated that residues corresponding to E192_ND1_, E143_ND1_, and D66_ND3_ are part of a larger, strongly coupled residue cluster, within which protonation states are highly anticorrelated and protons are effectively shared. This supports the physical relevance of a simulation framework employing a single mobile excess proton. In the same study, the residue corresponding to E34_ND4L_ was assigned to a P-side cluster with limited connectivity to the central cluster, indicating that proton transfer across the D66_ND3_–E34_ND4L_ junction is intrinsically unfavorable under equilibrium conditions. This conclusion is consistent with our free-energy results, which identify this region as a substantial barrier to proton transfer, while further showing that proton-induced reorganization of the hydration network can transiently alleviate this barrier and enable short-lived connectivity between D66_ND3_ and E34_ND4L_. From an electrostatic perspective, proton transfer along this direction increases the net negative charge within the central cluster, which may account for the free-energy increase of this process. In the context of mechanistic models that propose sequential injection of two protons into the E-channel, protonation of an additional residue within the central cluster—such as E68, which is sufficiently proximal to D66 but not directly involved in the PT pathway—could compensate the increased net negative charge and thereby reduce the associated free-energy increase. The influence of such coupled changes in protonation states on proton transfer energetics remains an open question and warrants further investigation.

While previous studies have suggested possible proton transfer pathways in Complex I,^6,17,28,30,32^ they have not captured the hydration-dependent behavior of the D66_ND3_–E34_ND4L_ region, which we address here using extended-sampling MS-RMD and water wire connectivity analysis. Although possible transient water network in this region has been reported in some experimental studies,^20,26,28,46,47^ those measurements provide time-averaged water densities and cannot quantify how an excess proton dynamically and transiently reshapes hydration and facilitates transfer. A recent QM/MM study likewise reported proton transfer across the E192–E143–D66–E34 segment, supporting the view that this region constitutes the principal conduit in the E-channel.^32^ Their analysis primarily examined how variations in protonation states modulate the proton-transfer energetics. However, with QM/MM sampling limited to only a few picoseconds per umbrella-sampling window, the water within the channel is virtually immobile on that time scale, making it unlikely to capture the excess proton-induced hydration dynamics. By employing nanosecond time scales and a quantitative analysis of water-wire connectivity, our MS-RMD simulations can capture these dynamics and delineate their thermodynamic consequences. It is also worth noting that recent advances in SCC-DFTB–based QM/MM methods,^53–55^ together with emerging machine-learning strategies for improving semiempirical Hamiltonians,^56,57^ suggest that future QM/MM approaches may achieve the longer-time scale sampling needed to serve as a useful complement for studying hydration-coupled PT dynamics in Complex I.

## Conclusions

In this work, we performed a quantitative free-energy and kinetic analysis of proton transfer along the ND1–ND4L segment of respiratory Complex I using MS-RMD simulations combined with water wire connectivity analysis. We have demonstrated that PT through the ND1-ND4L subunits in Complex I is coupled with local hydration, and that the excess proton itself promotes hydration in otherwise dry regions. Our free energy calculations reveal a substantial kinetic barrier in the D66_ND3_–E34_ND4L_ segment, which may serve as a regulatory gate in the overall proton pumping mechanism. These results suggest that hydration is not just a passive environmental factor, but an active component of proton gating and subsequent PT. This work also highlights the importance of treating hydration as a dynamic variable in mechanistic studies of PT in bioenergetic complexes and provides a computational framework that can be extended to other proton transporting proteins.

## Methods

### Classical MD and MS-RMD Simulation Details

The initial configuration was prepared from the cryo-EM structure of active *Mus musculus* Complex I [PDB ID: 8OM1].^47^ The protonation states of titratable residues were initially assessed using PROPKA.^58^ Residues with nonstandard protonation states were assigned based on consistency with prior study of active mouse Complex I.^29^ The entire subunits of Complex I were placed in a 1:2:2 of cardiolipin/POPC/POPE membrane and solvated with ∼450k TIP3P water molecules. 150 mM NaCl ions were added to neutralize the charge. FMN and Fe–S clusters were placed in the same position of the original structure, and the position of ubiquinone was modeled to be located at the entrance of E-channel, as identified in previous studies.^4,31,59–61^ The CHARMM36m (CHARMM36) force field was used for the protein (lipids). For FMN, Fe–S clusters, and ubiquinone, CHARMM style parameters were implemented from ref 62–64 respectively. All Fe–S clusters were modeled in their oxidized states, corresponding to a post–electron-transfer configuration in which the electrons have been transferred to the quinone. This choice reflects a simplified redox state intended to isolate proton transfer dynamics following quinone reduction, rather than to represent the full redox cycle of Complex I. To get the equilibrated structure, 1 μs of MD simulation in constant NpT ensemble at 310 K was done with the GROMACS^65^ simulation package with GPU acceleration. The protein backbones were fixed to their original coordinates for the first 300 ns of the simulation to relax the lipid conformation without disrupting protein packing. Subsequently, the position restraint was removed for the remaining 700 ns of the simulation.

After the classical MD simulation, we calculated the water wire connectivity (ϕ), as described in the following section. To ensure sufficient hydration, the configuration with the highest ϕ value within the last 100 ns of the simulation was selected and converted into the MS-RMD initial structure. MS-RMD simulation was done with RAPTOR^34^ module implemented in the LAMMPS^66^ MD package. In MS-RMD simulation, all the TIP3P waters are replaced by MS-EVB 3.2 ^67^ waters and the selected residues, E202_ND1_, E227_ND1_, E192_ND1_, E143_ND1_, D66_ND3_ and E34_ND4L_, were modeled as EVB-active, using the parameters reported in ref 42,43 . To ensure that only a single reactive excess proton was present in the system, the EVB-active residues were initialized in their deprotonated states, allowing them to participate in proton transfer events exclusively through interaction with the excess proton. In MS-RMD, an empirical valence bond (EVB) Hamiltonian is constructed using the predefined protonation states of water molecules and amino acid residues, and is diagonalized at each time step. The corresponding eigenvector coefficients determine the instantaneous delocalization of the excess proton among the EVB states. Full details of the MS-RMD are provided in the Supporting Information.

To calculate the PMF along ϕ without excess proton, we performed eight walkers well-tempered metadynamics simulation^68,69^ with LAMMPS and PLUMED simulation packages. Gaussian hills were deposited every 1,000 steps with a height of 0.6 kcal/mol and a width (σ) of 0.02 along the ϕ. The simulations were carried out at 310 K with a bias factor of 35. The walkers shared the biasing potential by reading hill files every 100 steps, allowing enhanced sampling of the free energy landscape. Each walker was simulated for 7.5 ns, resulting in a total simulation time of 60 ns across 8 walkers.

### Collective Variables

In this study, we introduce two CVs to show how the transport of excess protons depends on the hydration of the channel: the water wire connectivity (ϕ and ϕ*) and the curvilinear PT path CV (ξ*_PT_). The water wire connectivity, ϕ, is calculated as follows

1 $$\phi ={\left(\prod_{i=1}^{N-1}f_{i,i+1}\right)}^{1/\left(N-1\right)}$$

Here *f*_*i*,*i*+1_ is an average of the water occupancy of *i*th and (*i* + 1)­th node. As shown in the equation, ϕ is the geometric mean of the average occupancies of all node pairs along the path. In the local water wire connectivity, ϕ*, the occupancy of each node is weighted according to its distance from the excess proton. As a result, ϕ* reflects the water arrangement in the immediate vicinity of the excess proton.

The path CV is a geometrical path function calculated as follows

2 $$\xi_{\mathrm{PT}\prime }=i_{2}+sign\left(i_{2}-i_{1}\right)\cdot \frac{\sqrt{{\left(v_{1}\cdot v_{3}\right)}^{2}-|v_{3}{|}^{2}\left(\left|v_{1}{|}^{2}-\right|v_{2}{|}^{2}\right)}}{2|v_{3}{|}^{2}}-\frac{v_{1}\cdot v_{3}-|v_{3}{|}^{2}}{2|v_{3}{|}^{2}}$$

Here, **v**_**1**_ and **v**_**2**_ are the vectors connecting from the excess proton to the closest and the second closest nodes, respectively, while *i*_1_ and *i*_2_ are the indices of the nodes. **v**_**3**_ denotes the vector connecting from the closest node to the second closest node. By its definition, ξ_*PT*_ is unitless. For clarity and ease of interpretation, we introduce a shifted and rescaled path CV, ξ*_PT_, in which the node closest to D66_ND3_ is set to zero, the direction toward E34_ND4L_ is defined as positive, and the CV is rescaled to yield a length scale equivalent to Å. The detailed definition of both CVs can be found in the Supporting Information.

### Umbrella Sampling and WHAM

Umbrella sampling (US) was performed with the modified version of PLUMED v2.4.^33,70^ The system was integrated with a 1 fs time step and the Nose-Hoover thermostat in the constant NVT ensemble at 310 K. For the 2D-US simulations of ξ*_PT_ and ϕ*, a total of 130 US windows were used, with each window running for 1–2 ns, resulting in a total simulation time of over 200 ns. Harmonic force constants of 2500 kcal/mol and 10–30 kcal/mol were applied to ϕ* and ξ*_PT_, respectively. For the 1D-US simulation of the PT path CV (ξ*_PT_), 24 windows were used, with each window running for 1–1.5 ns, and force constants ranging from 10–30 kcal/mol were applied. The initial configuration for each window was created by dragging the excess proton along the path. The PMF of PT along the path was computed using the weighted histogram analysis method (WHAM).^71^ To ensure the convergence of the PMF, we performed block averaging for each umbrella sampling window. For the 2D-US calculations, the PMF was averaged over the last four of eight blocks. For the 1D-US calculations, the average was taken over the last three of six blocks.

## Supplementary Material



## Notes

Descriptions of the simulation and free energy calculation methods, and the mathematical details of the collective variables (PDF)
